# Prior Neurosurgery Decreases fMRI Estimates of Language Laterality in Patients with Gliomas within Anterior Language Sites

**DOI:** 10.3390/jcm10071491

**Published:** 2021-04-03

**Authors:** Monika M. Połczyńska, Bryan Ding, Bianca H. Dang, Lucia Cavanagh

**Affiliations:** 1 Department of Psychiatry and Biobehavioral Sciences, David Geffen School of Medicine at UCLA, University of California, Los Angeles, CA 90095, USA; biancahd@ucla.edu (B.H.D.); LCavanagh@mednet.ucla.edu (L.C.); 2 Department of Statistics, University of California, Los Angeles, CA 90095, USA; bryanding@ucla.edu

**Keywords:** brain tumor, glioma, previous surgery, laterality index, language dominance, fMRI, Broca’s area, Wernicke’s area

## Abstract

The impact of previous surgery on the assessment of language dominance with preoperative fMRI remains inconclusive in patients with recurrent brain tumors. Samples in this retrospective study included 17 patients with prior brain surgery and 21 patients without prior surgery (38 patients total; mean age 43.2, SD = 11.9; 18 females; seven left-handed). All the patients were left language dominant, as determined clinically. The two samples were matched on 10 known confounds, including, for example, tumor laterality and location (all tumors affected Brodmann areas 44/45/47). We calculated fMRI language dominance with laterality indices using a whole-brain and region of interest approach (ROI; Broca’s and Wernicke’s area). Patients with prior surgery had decreased fMRI language dominance (*p* = 0.03) with more activity in the right hemisphere (*p* = 0.03) than patients without surgery. Patients with prior brain surgery did not display less language activity in the left hemisphere than patients without surgery. These results were replicated using an ROI approach in the affected Broca’s area. Further, we observed no differences between our samples in the unaffected Wernicke’s area. In sum, prior brain surgery affecting Broca’s area could be a confounding factor that needs to be considered when evaluating fMRI language dominance.

## 1. Introduction

Several confounds have been shown to affect the estimates of language dominance in individuals with brain tumors around the language cortex of the language-dominant hemisphere, as shown by presurgical functional magnetic resonance imaging (fMRI). These confounds include, for example, tumor grade, tumor size, tumor location (i.e., anterior versus posterior language sites), age of tumor onset (e.g., pediatric versus adult), history of seizures, and presence of aphasia [1,2,3,4,5,6,7]. All of these variables need to be taken into consideration to correctly interpret the results of preoperative language fMRI [8,9]. An inaccurate assessment of fMRI language dominance in patients with brain tumors could increase the risk for surgery-induced impairment of language [6]. In patients with recurrent brain tumors, a history of prior brain surgery may be another significant confound. However, the scarce literature to date has been inconclusive regarding the impact of previous surgery on the assessment of language dominance with preoperative fMRI in patients with recurrent brain tumors [7,10,11,12,13]. 

Prior brain surgery can obscure observed language activations in fMRI in several ways. For example, brain tumor surgery can result in lower language activation due to dropouts in signal and image distortions (e.g., from titanium plates) when pre- and postoperative fMRI is compared [10,12,14]. A re-growing tumor may also infiltrate primary language sites and obliterate activations in these structures [10]. A few studies have directly compared fMRI language dominance in patients with brain tumors with and without a history of prior surgery. These studies have not found any systematic changes in language organization before and after brain surgery. For example, Kristo et al. [13] reported increased activation patterns in patients after brain surgery, but these changes were noted primarily in regions proximal to the surgical resection. The authors asserted that the alterations in the fMRI signal resulted from a (structural) postoperative shift rather than perilesional functional reorganization. At the same time, whole-brain alterations in the amplitude of language activation were frequently observed in individual patients [13]. Peck et al. [10] compared language laterality in patients with brain tumors with and without prior surgery and found no significant differences between the two groups. The authors applied both whole-hemisphere and region of interest (ROI) approaches to calculate LI values. Thus, those studies did not provide evidence that fMRI language laterality can be affected following brain surgery.

Nevertheless, several other studies have compared pre- and postoperative language organization in the same patients and found that it can (further) reorganize due to functional compensation, as indicated by fMRI [11] and magnetoencephalography [15,16]. For instance, Gębska-Kośla et al. [11] compared pre- and postoperative fMRI language dominance in patients with low-grade tumors around Broca’s area and those with tumors affecting Wernicke’s area. Among patients with tumors around Broca’s area, the authors found significantly increased activity in the non-affected right homolog of Broca’s region.

An essential limitation of the valuable research to date has been the lack of well-matched control samples. An appropriate control in examining fMRI language dominance in patients with prior surgery would include patients without prior surgery, matched based on all known confounding variables. For example, in the research carried out by Kristo et al. [13], patients with anterior and posterior brain tumors in the language-dominant left hemisphere were compared with individuals with tumors in the non-language-dominant right hemisphere, but these tumors were not matched for within-hemisphere location. Peck et al. [10] who examined fMRI language dominance using a whole hemisphere approach and an ROI approach (Broca’s area) in patients with (*n* = 16) and without previous surgery (*n* = 10) had only between two and three patients with tumors affecting the anterior language areas per group. The remaining tumors impacted temporal and parietal regions, and there was one case of an insular tumor. Thus, it was not possible to determine the impact of prior brain surgery within the anterior language sites. Further, while Kristo et al. [13] examined only patients with low-grade tumors, Peck et al. [10] did not provide information on tumor grade among their patients. Moreover, none of the studies seemed to match their participants on other potential confounds, such as tumor volume, aphasia presence, or the history of seizures.

As such, this research aims to determine if and how prior brain surgery distorts the assessment of language dominance with clinical fMRI, independent of other confounds known to impact laterality. Previous studies have demonstrated that the anterior language sites, as compared with other areas of the language cortex, including Wernicke’s area, are particularly susceptible to the disruption of fMRI language dominance following a brain tumor [2,7]. Thus, this study focused on the inclusion of patients with brain tumors affecting Broca’s area. Based on prior studies investigating changes in language dominance among patients with brain tumors [11,12], we hypothesized that language dominance as assessed with fMRI will be less robust in patients with prior surgery compared to patients with brain tumors who had not yet undergone brain surgery. More specifically, we expected less language activity in the left language dominant hemisphere and more activity in the right hemisphere in patients with prior surgery than in patients with no prior surgery.

## 2. Materials and Methods

### 2.1. Subjects

This research was approved by the UCLA Institutional Review Board. We retrospectively reviewed the data of over 1300 patients who underwent preoperative fMRI at UCLA between 2009 and 2020. The database included patients (both adult and pediatric) diagnosed with conditions such as (but not limited to) brain tumors, epilepsy, and arteriovenous malformations. Patients eligible for inclusion in this study were monolingual, native speakers of English with gliomas affecting Broca’s area (Brodmann areas 44, 45, and/or 47). Among those eligible, only patients with sufficient medical data were included in the final sample in order to allow careful sample matching. Two groups were identified: (1) a target group of 17 patients with a history of prior surgery who received preoperative language fMRI due to glioma recurrence, and (2) a control group of 21 patients with no prior history of brain surgery who received preoperative language fMRI before their initial surgery. The final sample thus included 38 individuals (mean age 43.2, SD = 11.9; 18 females; seven left-handed; Table 1). Time since surgery in the patients with prior surgery ranged between four to 128 months (mean 43.5 months, SD = 42.1).

All of the patients were left hemisphere dominant for language, as determined clinically. This clinical determination was based on the results of a neurocognitive assessment, language fMRI read performed by clinical neuropsychologists (who were blind to this study), and—in 13 cases (34.2% of the total sample)—direct cortical stimulation (all conducted before our data analysis). Therefore, the retrospective clinical determination of the patients’ language dominance could be different from the indices of language fMRI laterality calculated for this study.

### 2.2. Controlling For Confounds in Sample Matching

**Figure 1:**
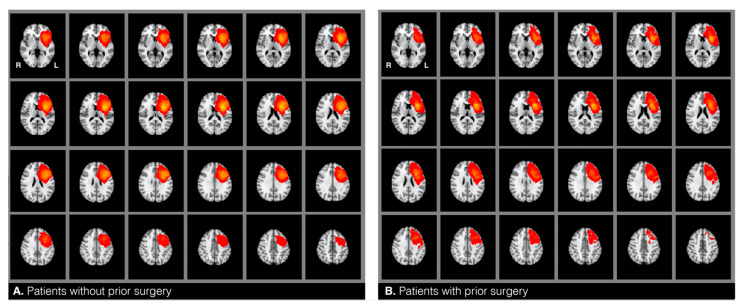
Heatmaps representing the location of gliomas in patients without (**A**) and with (**B**) prior surgery.

The target and the control groups were statistically compared based on twelve variables that can potentially confound fMRI language dominance [1,2,3,4,5,6,7]. These included: (1) glioma hemisphere, and (2) location—all patients included in this study had a brain tumor around the left inferior frontal gyrus (see heat maps in Figure 1), (3) glioma volume—mean tumor size was calculated for each group by drawing tumor masks (see below), (4) glioma grade—i.e., low versus high grade, with additional consideration of the frequency of distribution of grades I−IV, and (5) glioma type [17]. Glioma grade and type were determined through record review. All patients were diagnosed with type I tumors, which are diffuse astrocytic and oligodendroglial tumors (gliomas) [17]. We compared the frequency of the distribution of the following glioma types between the two samples: glioblastoma, anaplastic astrocytoma, oligoastrocytoma, and oligodendroglioma. We also compared the samples based on (6) the presence of aphasia and (7) the history of seizures. The two factors were accessed from information gathered during the patients’ fMRI visit and/or from record review indicating aphasia diagnosis by a neuropsychologist. Additionally, the samples were compared on the following demographic variables: (8) handedness, (9) age, and (10) gender. These were identified from patient reports and record reviews. To assess handedness, patients were asked to indicate with which hand they wrote. This information was checked against record reviews for concordance. Finally, we also reviewed how many patients underwent (11) chemotherapy and (12) radiotherapy across the two groups. Chi-square and Fisher’s exact tests were used to analyze categorical variables when appropriate. An independent samples t-test was used to analyze continuous mean age differences. The Mann–Whitney-U test was used to compare glioma volumes between the two samples.

### 2.3. Procedures

In this study, we followed procedures that we used in our prior research [18]. The patients participated in a single clinical fMRI session. Before the scan, they were screened for aphasia by answering detailed questions about their subjective language expression and comprehension abilities. They also answered questions about their handedness and seizure history. Next, the patients practiced sample items from three fMRI language tasks before being placed in the scanner. The practice items were different from those presented to the subjects in the scanner. Each task performed in the scanner consisted of three items (task details are presented below). Whenever possible, the subjects completed two runs consisting of three language tasks each while inside the scanner. In some cases, patients only completed one run when the time was constrained because of other factors, such as the patient becoming too tired to complete the second run of the language tasks.

Scans were completed in a 3T Prisma or Allegra scanner (20- and 12-channel head coils, respectively). Patients who were scanned with each scanner were balanced across the two groups. Specifically, in the group with prior brain surgery, five subjects (27.8%) were scanned with the Allegra scanner and thirteen (72.2%) were examined with the Prisma scanner. In the group with no prior surgery, six patients (28.6%) were scanned using the Allegra scanner and fifteen individuals (71.4%) underwent their exam using the Prisma scanner. The following echo-planar image parameters were applied: TR = 2500 ms, TE = 35 ms, 90° flip angle, 28 slices, 90 volumes, voxel size 3.1 × 3.1 × 3 mm^3^, matrix dimensions 200 × 200 mm^2^, field of view = 200 mm. The following parameters were applied for T2 images: TR = 6670 ms, TE = 58 ms, 90° flip angle, voxel size 0.5 × 0.5 × 3 mm^3^, matrix dimensions 263 × 350 mm^2^, field of view = 200 mm, turbo spin echo, and generalized autocalibrating partial parallel acquisition (GRAPPA, acceleration factor = 2).

Language activations were mapped using a conjunction analysis of three language tasks. In the first task (object naming), the subjects were directed to silently name a black-and-white, concrete object that they saw on a screen (e.g., a banana). In the second task (reading or visual responsive naming), the participants were instructed to silently read a phrase (e.g., “wear them on feet”) and think of the object being described. During the third task (auditory responsive naming), the patients were asked to listen to a phrase (e.g., “color of the sky”) and think of the object being described [19,20]. Each of the tasks involves a different modality to trigger language activations [21,22]. All the tasks used a block design that had the following procedure: 1 s of a written cue (e.g., “think of the name”), followed by a 10 s activity block with three trials, or a rest block (i.e., a crosshair on a screen). There were 11 task blocks and 12 rest blocks. The total duration per task was four minutes.

### 2.4. Analyses

Our analyses were similar to those described in Połczyńska et al. [7].

#### 2.4.1. Analysis of fMRI Data

A custom image analysis software for clinical fMRI built at UCLA was used to pre-process and analyze the data. The software converts raw data in the digital imaging and communications in medicine (DICOM) format to image files (.bshort) and files with statistical maps (.bfloat). The latter files contain information about the correlation of the paradigm convolved on the hemodynamic response function for each voxel. The data is visually inspected for various potential artifacts (e.g., ring artifacts, excessive noise, radio frequency interference, steal artifacts, etc.). Patients typically perform each of the three language tasks twice, which allows for a selection of the best run for each task to perform a conjunction analysis. Motion correction is rarely done because it can warp the data, while changes of millimeters in locations of voxels can make a difference in neurosurgical planning. If there is a clear motion pattern within a given task (e.g., the head position shifted towards the end of the task), selected repetition times are excluded from the analysis. Activations related to the language tasks were determined using Pearson’s Correlation Coefficient (see Benjamin et al. for details) [22]. Data were smoothed with a two mm Gaussian kernel. We convolved a regressor that included the expected time series with a hemodynamic response function and then computed the correlation of the observed activity with expected activity. We checked the data for quality. In cases where the subjects completed two runs of the language tasks, we selected the more superior quality run for use in the subsequent analyses. We used an initial correlation threshold of *r* = 0.2. This threshold was subsequently adjusted until we identified an optimal representation of the language network.

We applied the conjunction of the language maps resulting in the most superior representation of the language function. This approach yielded significance values of *p* < 0.000123 (0.05^3^). The approach is based on the Bayes theorem [23], according to which the joint probability of two or more events occurring simultaneously by change is the product of each prior probability. We set the prior probability as less than 0.05—the probability of having a pixel activated randomly in a single spot, so the joint probability is less than the product of each prior probability. This approach demands that each voxel itself be significant [24]. The approach effectively eliminated any activation that is not specific to language function (e.g., visual activation in the reading responsive naming task). The approach is systematic, valid, and reliable in comparison with other assessments of language dominance [22].

#### 2.4.2. Glioma Identification

Individual glioma borders were based on the changes in signal intensity on T2. With the T2 boundaries, we manually drew masks around the patients’ glioma on T2-weighted MRI scans in FMRIB Software Library View (FSLView) and additionally used T1 images for reference. A second (senior) investigator reviewed each of the masks for accuracy. The tumor masks included the center of the glioma, dense surrounding edema (if any), and any previous resection cavity, as all of these lesion characteristics can distort language signals. T2 images were skull-stripped using a “bet” command. Using linear regression (12 degrees of freedom), the betted images were transformed to normalized brain space (Montreal Neurological Institute, MNI, 152 T-1 weighted, 2 mm). A “flirt” command was applied to transform tumor masks from individual space to the MNI space. Next, an “fslstats” command was used to calculate tumor volume of the transformed tumor masks. We reviewed the distribution of the glioma masks by overlaying all the masks within each of the two samples and thereby generated heat maps for each of the two groups in the MNI space (Figure 1). Finally, also using the MNI space, we calculated glioma volume using the tumor masks (fslstats).

#### 2.4.3. Calculation of Language Dominance with the Language Laterality Index

The laterality index measure was used to evaluate language dominance during the fMRI language tasks. A standard approach to calculating the laterality index is expressed in the formula L − R/L + R, where results can range from +1 (indicating strong left hemisphere dominance) to −1 (indicating strong right hemisphere dominance) [25]. The thresholded voxel counts that survived the conjunction analysis were applied to determine the laterality index with the whole brain, as well as four ROIs that were derived functionally. Specifically, the ROIs included (1) left Brodmann’s areas (BAs) 44, 45, and 47 (Broca’s area), (2) left BA 22/39/40 (Wernicke’s area), (3) the right homolog of Broca’s area, and (4) the right homolog of Wernicke’s area. We will refer to (1) as the left anterior language areas and (2) as the left posterior language areas. Based on the previous literature, as well as our clinical practice, we know that language tasks involving production recruit the left anterior language sites (Broca’s area). In contrast, tasks that require language comprehension engage the left posterior language regions (Wernicke’s area) [18,19].

The ROIs were delineated individually for each subject using their individual space in a .bfloat format. Functional language tasks resulted in areas of blood oxygen level-dependent activations for each of these four regions. Activations in these regions were thresholded individually for each subject (threshold range = 0.1−0.22). Activity for each functional ROI was manually highlighted. Using a number of pixels (npix) function, a thresholded number of active pixels within the highlighted functional ROI was displayed.

Structural displacements of functional areas caused by tumors were taken into consideration during the delineation of the ROIs. In some cases, larger brain tumors pushed functional tissue away from where Broca’s area was expected. Activations in those areas were included in the analyses. The structural displacements were identified by a senior investigator who additionally incorporated notes from clinical fMRI reports that always stated such displacements.

#### 2.4.4. Evaluation of the Study Hypothesis

We examined the influence of prior surgery on fMRI language dominance with a series of independent samples t-tests. Voxel counts and language laterality values were compared between patients with and without prior surgery using both a whole-brain approach and an ROI approach.

For the whole-brain approach, we first assessed how prior surgery impacted the total number of voxels active during the language tasks in each hemisphere. Based on these numbers, we calculated fMRI language laterality values for patients with and without surgery. Finally, we compared these laterality values between the two groups.

For the ROI approach, we compared the number of active voxels in Broca’s and Wernicke’s areas and their homologs in the right hemisphere in patients with and without surgery. We then used these numbers to calculate language laterality values in each of the two ROIs. Finally, we compared the language laterality values in these ROIs between the two groups.

We used an unpaired t-test to compare mean values of language laterality based on the number of active language voxels in the following regions: (1) a whole hemisphere versus Broca’s area, (2) a whole hemisphere versus Wernicke’s area, and (3) Broca’s area versus Wernicke’s area. We conducted the analyses for each of the two patient groups separately. Based on our previous study [7], Wernicke’s area can serve as a control region for calculating non-disrupted language dominance because it does not appear to be affected by the presence of a brain tumor in Broca’s (or Wernicke’s) area.

Since each of the two patient samples included low- and high-grade glioma cases, we used unpaired t-test analysis of tumor grade and fMRI language laterality values between the two samples. The analysis aimed to determine whether glioma grade was associated with different language laterality values. We additionally utilized the Pearson coefficient test and conducted a few regressions to analyze the effect of time between the prior surgery and the fMRI exam reported here on language laterality values. Finally, we applied an unpaired t-test to compare the average correlation threshold used in each group.

## 3. Results

### 3.1. Sample Matching

The two patient samples did not significantly differ on ten out of twelve potentially confounding variables, including glioma hemisphere, location, volume, grade, type, the presence of aphasia, the history of seizures, handedness, age, and gender. Most patients with prior surgery underwent chemotherapy (χ^2^(1, *n* = 27) = 2.76, *p* = 0.000) and radiotherapy (χ^2^(1, *n =* 27) = 14.85, *p* = 0.000), while none of the subjects without prior surgery had received these treatments at the time of their fMRI exam. Of note, chemo/radiotherapy history information was missing for one of the 17 patients with prior surgery and nine of the 21 patients with no surgery. We conducted a Fisher’s exact test to examine the relationship between chemotherapy and radiotherapy. The results of the test demonstrated that there was no relationship between the two types of treatment. For details on sample matching, see Table 1.

### 3.2. The Impact of Brain Surgery on Active Voxel Counts and Language Laterality

**Figure 2:**
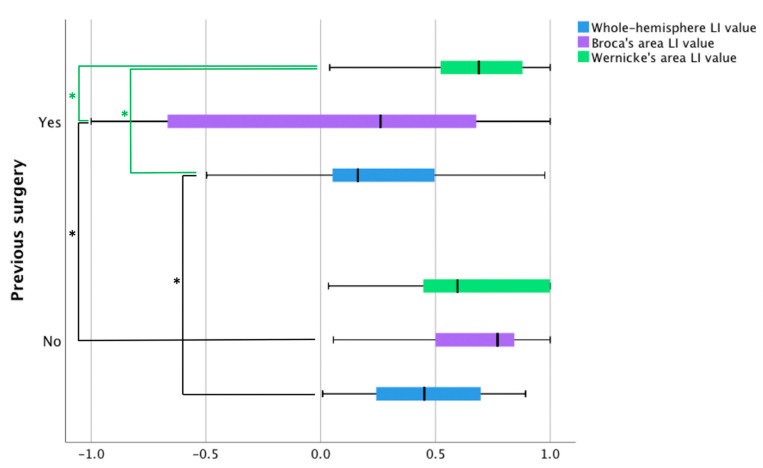
Language laterality values in patients with and without prior surgery based on three fMRI language tasks. The blue bars represent fMRI laterality values based on a whole-hemisphere approach. The remaining two bars illustrate laterality values using an ROI approach in Broca’s area (purple bars) and Wernicke’s area (green bars). LI = language laterality. Black lines with significance stars represent between-group differences, whereas green lines with significance stars show within-group differences.

The average correlation thresholds of the two patient samples used in the fMRI analyses were not significantly different (*t*(21) = 0.502, *p* = 0.62 (95% confidence interval from −0.02 to 0.03). Using the whole-brain approach, it was found that the mean number of voxels active during the language tasks in the left hemisphere was not significantly different between the patients with and without prior surgery (Figure 2). Patients with prior surgery had more active voxels in the right hemisphere (*n* = 17, *M* = 218.88, *SD* = 131.61) compared to patients with no prior surgery (*n* = 21, *M* = 128.38, *SD* = 111.01), *t*(26) = −2.3, *p* = 0.03 (95% confidence interval from −170.3 to −10.7). As the patients with prior surgery displayed more robust activity in the right hemisphere, their language laterality values were lower (*n* = 17, *M* = 0.24, *SD* = 0.36) than patients with no prior surgery (*n* = 21, *M* = 0.47, *SD* = 0.25), *t*(36) = 2.23, *p* = 0.03 (95% confidence interval from 0.02 to 0.42).

Within-group comparisons of mean values of fMRI language laterality generated no significant results for the patients with no prior surgery. In patients with prior surgery, we found significant differences between fMRI language dominance values based on Wernicke’s area compared to the values based on Broca’s area (*p* = 0.002) and a whole hemisphere (*p* = 0.006). There was no significant difference between dominance values derived from the number of active voxels in Broca’s area and a whole hemisphere (Figure 2).

**Figure 3:**
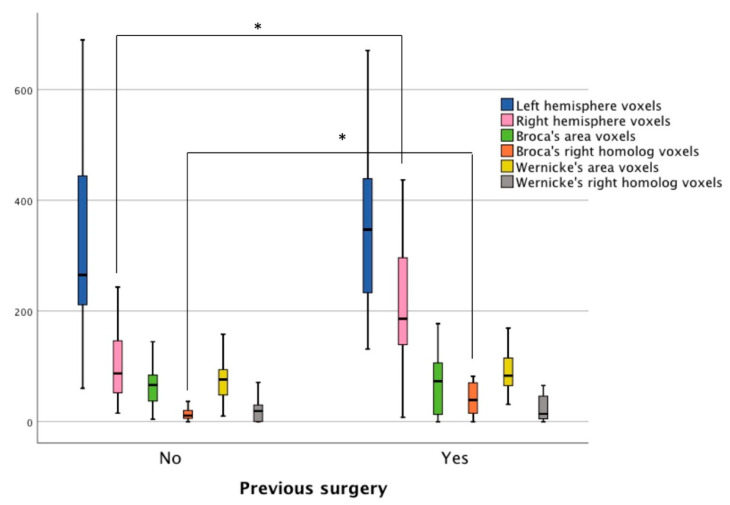
The number of active voxels in patients with and without prior surgery based on three fMRI language tasks. The navy blue and pink bars represent voxel counts using a whole-hemisphere approach. The remaining bars display voxel counts in four ROI: Broca’s area (green bars), the right homolog of Broca’s area (orange bars), Wernicke’s area (yellow bars), and the right homolog of Wernicke’s area (grey bars). Stars (*) represent statistically significant differences.

Using the ROI approach, it was observed that the mean number of voxels active during the language tasks in Broca’s area was not significantly different between the two samples. The mean number of active voxels within the right hemisphere homolog of Broca’s area was higher in the patients with prior surgery (*n* = 17, *M* = 45.94, *SD* = 39.23), compared to the individuals with no prior surgery (*n* = 21, *M* = 9.52, *SD* = 23.18), *t*(36) = −2.58, *p* < 0.01 (95% confidence interval from −47.16 to −5.67). Due to the elevated activity in the anterior right hemisphere ROI, the patients with prior surgery had lower language laterality values in Broca’s area (*n* = 17, *M* = 0.06, *SD* = 0.70) than the patients with no prior surgery (*n* = 21, *M* = 0.56, *SD* = 0.47), *t*(36) = 2.6, *p* < 0.01 (95% confidence interval from 0.11 to 0.88). No significant differences were found between the groups for the mean number of voxels in Wernicke’s ROI or its right hemisphere homolog ROI. Language laterality values in Wernicke’s area were nearly the same for the two groups (Figure 3).

Finally, glioma grade (low versus high) did not significantly affect any of the language laterality values. Pearson coefficient test and several regressions showed no relationship between time since prior surgery on language laterality values.

## 4. Discussion

The current study examined the impact of previous brain surgery on language dominance as assessed by fMRI, independent of numerous known confounds. Our hypothesis confirmed that the patients with prior surgery in the dominant left hemisphere had lower language laterality values and more activity in the right hemisphere than patients who did not previously undergo brain surgery. Contrary to what we hypothesized, however, the patients with prior brain surgery did not display less language activity in the left hemisphere than the patients who did not have prior surgery. These results were replicated using an ROI approach in the affected Broca’s area. An important clinical implication of these findings is that prior brain surgery can be a confounding factor that needs to be taken into account when evaluating fMRI language dominance in patients with recurring gliomas around the inferior frontal gyrus. Further, there were no observed differences in the unaffected Wernicke’s area, as language laterality values were almost the same in both groups. Thus, we suggest that, among patients with prior surgery who have gliomas around Broca’s region, fMRI language dominance should be assessed using ROI within unaffected posterior language areas.

While prior research compared fMRI language dominance in patients with and without previous brain surgery [10,13], the studies did not control their samples for several important confounds, such as tumor location. It is possible that because of the considerable variations between their patient groups, the studies did not observe significant differences in fMRI language dominance. Concurrently, one of the reports [13] noted that—while there were no significant differences between samples on a group level—there were frequent changes in the amplitude of language activation in individual patients. In the current study, we were able to control for 10 factors known to distort fMRI language dominance. We found that patients with prior surgery had lower language laterality values and more activity in the right hemisphere than patients with no prior surgery.

The elevated activation in the right hemisphere during fMRI language tasks is in line with the results presented by Gębska-Kośla et al. [11], who examined fMRI language dominance before and after tumor resection. The authors found that individuals with gliomas affecting Broca’s area displayed increased activation in the non-affected right homolog of Broca’s area. They suggested that the elevated activation was due to functional compensation. This explanation appears feasible because the sample only included patients with low-grade gliomas. Functional redistribution is possible in patients with low-grade gliomas. Previous studies have shown that a low-grade tumor can gradually destroy Broca’s area, but language function can reorganize away from the tumor to other regions to minimize functional language loss [2,26,27,28]. While the functional redistribution frequently occurs within the affected hemisphere [29,30], it is not uncommon to see functional involvement of right hemisphere homologs of the lesioned structure [31,32].

The increased right hemisphere activity in our sample may have been caused by functional compensation but only in a subgroup of patients with prior surgery. Among the patients with prior surgery, nine individuals were diagnosed with low-grade gliomas and seven with high-grade gliomas. Among the nine patients with low-grade gliomas, four were diagnosed with aphasia. Among the patients with high-grade gliomas, all had aphasia. While high-grade gliomas and aphasia are not usually associated with functional compensation [31,32], the process might have taken place in the small group of five patients with low-grade gliomas with no aphasia diagnosis. Yet, we lack postoperative data for these patients to assess whether this activation was indeed compensatory. In the remaining patients, pseudo-reorganization may have been more likely. Pseudo-reorganization is a phenomenon that involves functional disinhibition of the contralesional hemisphere following a brain mass. It is more common in patients with more aggressive, high-grade gliomas. In these cases, fMRI language activations do not represent actual compensatory mechanisms, and language performance is often disrupted [33].

A recent report by our group [7] examined the impact of brain tumor location in the language-dominant hemisphere on fMRI language dominance. The study demonstrated that tumors around Broca’s region decreased fMRI language dominance independently of known confounds, while tumors around Wernicke’s area did not significantly disrupt fMRI language dominance. Both tumor groups were compared to cases of brain tumors affecting homolog sites in the non-language dominant hemisphere. In that study, we included both patients with and without prior surgery, although we ran additional analyses to assure that there were no differences between the two groups in fMRI language laterality values. The results of our current study confirm these prior findings in that: (1) brain tumors around Broca’s region decrease whole-hemisphere fMRI language laterality, and (2) language laterality in Wernicke’s region seems unaffected by left anterior tumors. These findings hold both for patients with and without previous brain surgery in the current study. While language laterality in the affected Broca’s region significantly decreased in the previous sample (which included patients with and without prior surgery), the current results revealed a decrease in language laterality only for the individuals with a prior history of surgery. By contrast, the patients with no prior surgery did not demonstrate decreased language laterality values in Broca’s region. In fact, their laterality values in Broca’s region were higher than the whole-hemisphere-based language laterality. A possible explanation for these findings may be that fMRI language laterality in Broca’s area significantly decreases following tumor resection and subsequent tumor regrowth. First-time tumors that have not been surgically removed may not considerably disrupt language laterality in this region. This interpretation would account for the robust language laterality in Broca’s area in the individuals with no surgery.

The findings reported in this study can inform clinical practice. The elevated activity in the right hemisphere in patients with prior surgery during language tasks can increase the risk of making false interpretations about language organization. There are at least two possible scenarios of how the activity could be misinterpreted. First, activity in right hemisphere could look like functional compensation, falsely suggesting that the right hemisphere has taken over (some) language functions of the left-dominant hemisphere affected by the tumor. Second, right hemisphere activity could give an incorrect impression about pre-morbid right hemisphere dominance in patients in whom dominance was not previously assessed [33,34]. Under both scenarios, a neurosurgeon could assume that surgery near Broca’s area is safe because the right hemisphere can subserve language function. Based on our results, as well as our prior findings [7], we thus suggest that in patients with prior surgery, evaluating fMRI language dominance using Wernicke’s area ROI can provide a more adequate estimate of language dominance. We demonstrated that fMRI dominance in Wernicke’s area is less likely to be affected by brain tumor than fMRI dominance calculated based on Broca’s area or whole-hemisphere activity. In addition, there were no significant differences in laterality values in Wernicke’s area between the two patient groups, which also suggests that fMRI language activity in this area is not disrupted by brain tumors in the anterior language sites. It should be noted, however, Wernicke’s region has been shown to be less lateralizing area than Broca’s region [35,36,37]. Therefore, fMRI estimates of language dominance based on activity in Wernicke’s area can be slightly lower in comparison to estimates based on a whole-hemisphere or unaffected Broca’s area. Further, we highlight the importance of conducting neuropsychological evaluations of language to complement the assessment of fMRI language dominance.

**Figure 4:**
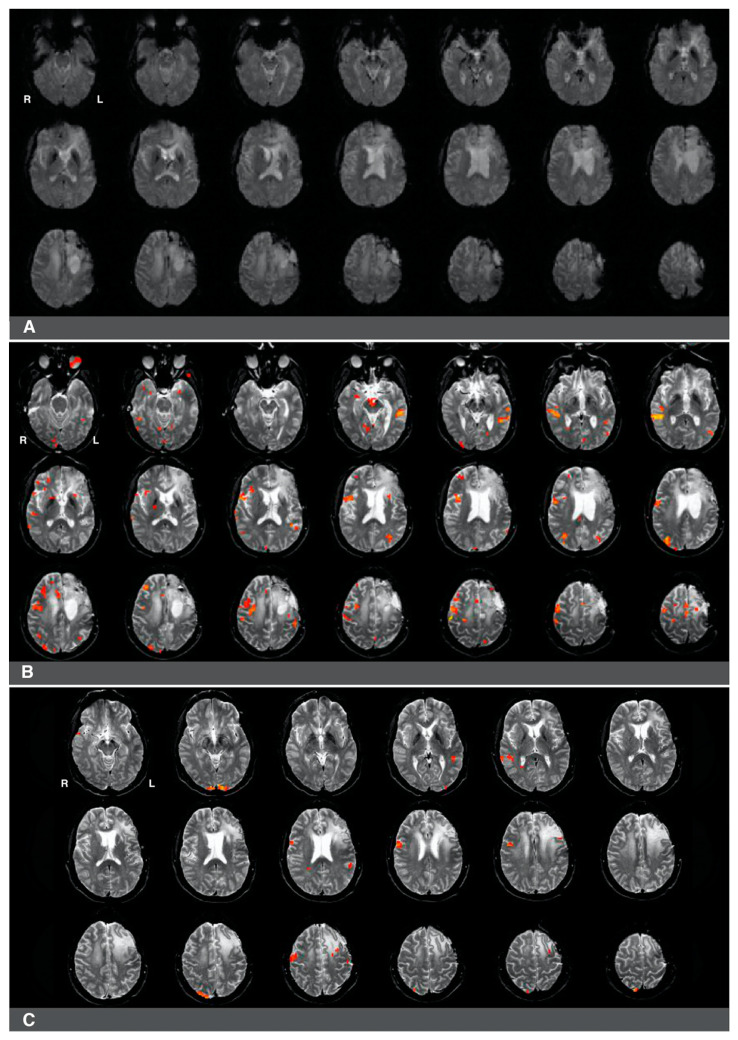
Right-hemisphere activations in a (clinically confirmed) left language-dominant patient with a high-grade glioma in the left hemisphere. Panel (**A**): a raw functional image with a notable absence of activation in the left frontal region corresponding to Broca’s area. The absence of activation may be related to artifact and edema from prior resection in the area. Panel (**B**): Language activations consistent with Broca’s area observed in the right hemisphere during pre-surgical fMRI. Panel (**C**): Prior fMRI from six months earlier indicates Broca’s representation in the left hemisphere.

**Figure 5:**
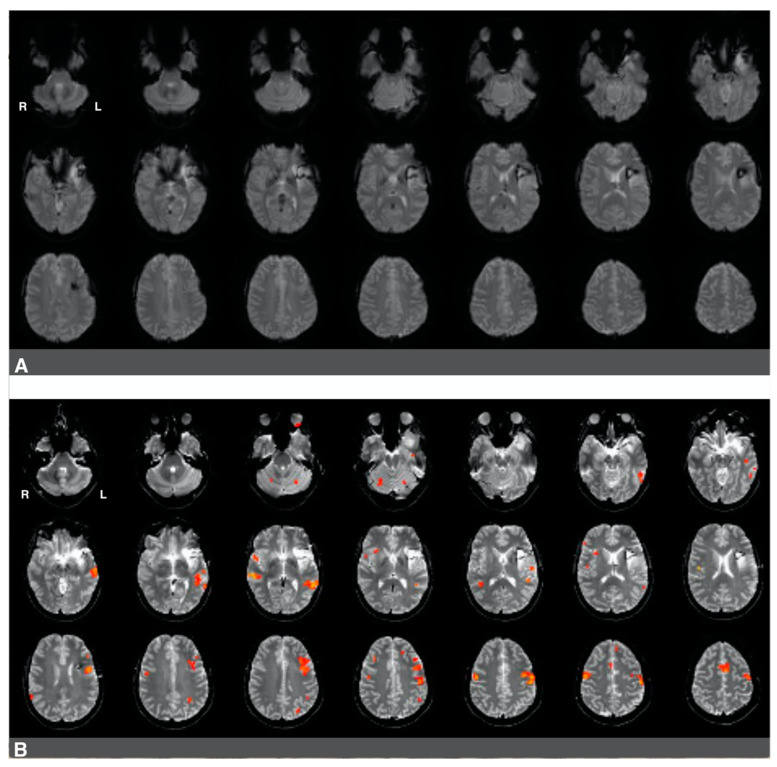
Predominantly left hemisphere activations in a patient with a low-grade glioma in the left hemisphere. Panel (**A**): a raw functional image with a notable absence of activation in the left frontal region corresponding to Broca’s area. The absence of activation may be related to artifact and edema from prior resection in the area. Panel (**B**): Language activation consistent with Broca’s area during pre-surgical fMRI. Activations in the right hemisphere were much less significant in this patient.

While language laterality values were significantly lower in patients who underwent prior surgery (whole-hemisphere LI and Broca’s area LI), we noted a high degree of individual heterogeneity in this group in terms of the range of language laterality values observed. This considerable variability suggests that these patients may be differentially affected by several factors associated with the previous resection (e.g., the presence of surgical hardware), glioma regrowth (e.g., glioma size, glioma grade, edema), and the amount of subsequent right hemisphere engagement in language. Signal artifacts constitute a particular challenge in interpreting the results of preoperative fMRI since activations in eloquent language sites can be obscured. In cases where surgical hardware and/or edema may impair fMRI activations, it is more challenging to evaluate whether right hemisphere activity during language tasks (if present) is compensatory in nature or whether it is caused by pseudo-reorganization (i.e., disinhibition of the right hemisphere) [8,11,16,33,34]. Figure 4 and Figure 5 present two cases that illustrate several challenges associated with interpreting the results of presurgical language fMRI in individuals who underwent a previous resection.

The first case was a 47-year-old, left-handed male with a diagnosis of a left frontal glioma. The patient was diagnosed with stage III oligoastrocytoma, and he suffered from significant aphasia. The patient underwent chemo- and radiotherapy and had a history of seizures. His fMRI language laterality based on the whole-hemisphere approach was −0.5. Language laterality was −1 using Broca’s ROI and 0.05 using Wernicke’s ROI. As shown in Figure 4, the eloquent cortex was identified in both the right and left hemispheres in this patient. There was a notable absence of activation in the left frontal region corresponding to Broca’s area that may be related to artifact and edema from prior resection in the area (Figure 4A). Language activations consistent with Broca’s area were observed in the right hemisphere in images four and seven of the second row and the first image of the third row (Figure 4B). Prior fMRI from six months earlier indicates Broca’s representation in the left hemisphere (Figure 4C, row two, slice five). The eloquent language cortex in this area was confirmed with speech arrest during awake mapping. Additionally, the patient experienced significant language decline in the last two months proceeding the latest clinical fMRI. Thus, while the activity in the right hemisphere seemed to have some compensatory effect on language function, the patient was not right hemisphere dominant for language, as the whole-hemisphere-based and Broca’s-based language laterality might suggest. Laterality index values obtained from Wernicke’s region appeared more reliable in this case.

The second case was a 37-year-old, left-handed female diagnosed with stage II astrocytoma. The patient suffered from mild aphasia. The patient underwent chemo- and radiotherapy and had a history of seizures. Language laterality based on the whole-brain approach was 0.46, and it was 0.65 using Broca’s ROI and 0.54 using Wernicke’s ROI. Activation consistent with Broca’s area was observed in Figure 5B, images one to four of the third row, immediately adjacent to the superior aspect of the lesion and prior resection site. Activations in the right hemisphere were much less significant in this patient. There was an artifact from the prior surgery in the area of the lesion, which is likely obscuring further activation in Broca’s area in the left hemisphere (Figure 5A).

Functional fMRI has become a non-invasive technique to map language functions pre-operatively. The method helps navigate neurosurgery and predict post-surgical risk to language functions [9,22]. Nevertheless, fMRI has its challenges that need to be considered when discussing the results of the current study. For instance, the technique may produce false positives (i.e., it can identify some areas as eloquent, whereas these regions only support but are not essential for intact language abilities) [22,38]. Therefore, intraoperative brain mapping remains the gold standard of identifying eloquent language sites that need to be preserved to avoid post-surgical language impairment [9,39].

This study has several limitations that should be considered when interpreting its results. The main limitation is associated with the retrospective design of this work, resulting in suboptimal documentation and incomplete medical records. Second, the study sample is relatively low. We carefully matched our patient groups based on the known variables that can disrupt language dominance values assessed with fMRI. This approach minimizes the effect of possible confounds on our findings, thereby addressing a major gap of prior research in this area. Third, it is a caveat that an independent clinical measure of language dominance was missing in some cases (i.e., 34.2% of the patients underwent electrocorticography). Forth, we included patients who were left-handed, which can be viewed as a limitation, although it is noted that the patient samples were balanced for the distribution of handedness. Moreover, since the sample in this study is a reflection of actual clinical referrals, we thought it was important to include left-handers, as this may bolster the external validity of the current findings. Fifth, we caveat that glioma boundaries were determined by the manual drawing of tumor masks. While this approach is not bias-free, we had a second (senior) investigator review each of the masks for accuracy. The locations of gliomas and glioma boundaries were examined using anatomical images. To minimize the source of bias, the masks were blind to fMRI data. Sixth, we lack data from postoperative language assessments that could help evaluate the nature of the elevated activity in the right hemisphere during the fMRI language tasks in patients with prior surgery (i.e., pseudo-reorganization/disinhibition versus functional compensation). Seventh, the approach comparing patients with and without prior surgery has its strengths and weaknesses. Another evident weakness is the lack of postoperative data on changes in the neural organization of language after glioma resection in each of the groups. Nonetheless, comparing well-matched samples using preoperative fMRI may help advance our understanding of the impact of prior brain surgery as a possible confound that could bias the assessment of language dominance. Eighth, our custom image analysis software did not allow us to directly compare the two patient samples on the amount of movement during the fMRI language tasks. However, we used Wernicke’s area as a control region, assuring equal BOLD responses across the two groups. Ninth, we applied linear regression that is not free of limitations because the structure of the brain may be warped by the tumor mass. We chose linear registration instead of non-linear registration because the linear registration MNI atlas (the atlas is provided by the FSL). In our experience, non-linear registration is particularly difficult with functional data because of the warping from functional to structural overcorrects, which creates a risk of anatomically unfeasible results. Given the presence of tumor pathology, we tested using a binary cost function. However, this did not yield improved registration results. Therefore, we used linear registration with 12 degrees of freedom, which FSL recommends for registering to linear MNI space [40]. Finally, calculation probabilities based on the Bayes theorem are not free of complications in neuroimaging. The true probabilities are affected by their neighbors due to spatial smoothing. Specifically, each voxel has the chance of being increased in magnitude by smoothing its neighbors, but equally, each voxel can be reduced in magnitude by smoothing with inactivated neighbors. The “true” probability is further complicated by the fact that these are spatial statistics. Therefore, the probability is the joint probability (product) of each prior pixel value, times the probability of occurring in a specific spatial location. However, this is offset essentially equally by the number of multiple comparisons (either each voxel, or after smoothing, each resolution element). Thus, it would be more accurate to say that the probability approximates the product of the prior probabilities that we set [24].

## 5. Conclusions

This study examined the impact of previous brain surgery on fMRI language dominance, independent of known confounds. We compared two groups of patients with left hemisphere language dominance who were diagnosed with brain glioma s around the inferior frontal gyrus: patients who had undergone prior glioma resection and had recurrent brain gliomas and patients who had not yet had initial brain surgery. We demonstrated that the patients with previous surgery had lower fMRI language laterality values and more activity in the right hemisphere than patients who did not undergo brain surgery. An important clinical implication based on these findings is that prior brain surgery can be a confounding factor that needs to be taken into account when evaluating fMRI language dominance in patients with recurring brain gliomas around the inferior frontal gyrus. Further, we observed no differences between our samples in the unaffected Wernicke’s area, with language laterality values being almost equal in both groups. Thus, we suggest that in patients with previous surgery and gliomas around Broca’s region, fMRI language dominance should be assessed using ROI within unaffected posterior language areas.

## Figures and Tables

**Table 1:** Biodemographic information for patients with and without prior surgery and missing variables.

Variable		Previous Surgery	No surgery	Total	Total Missing Variables
Number of patients		17	21	38	0
Gender	Females	9 (50%)	9 (50%)	18	
	Males	8 (40%)	12 (60%)	20	
	Total	17 (45%)	21 (55%)	38	0
Mean age		44.8	41.9		
Handedness	Right	13 (42%)	18 (58%)	31	
	Left	4 (57%)	3 (43%)	7	
	Total	17 (45%)	21 (55%)	38	0
Tumor grade					
	High grade	7 (41%)	10 (59%)	17	
	Low grade	9 (47%)	10 (53%)	19	
	Total	16 (44%)	20 (56%)	36	2
Tumor type					
	Glioblastoma	4 (33%)	8 (67%)	12	
	Anaplastic astrocytoma	7 (58%)	5 (42%)	12	
	Oligoastrocytoma	1 (20%)	4 (80%)	5	
	Oligodendroglioma	5 (56%)	4 (44%)	9	
	Total	17 (45%)	21 (55%)	38	0
Seizures					
	Yes	14 (54%)	12 (46%)	26	
	No	3 (25%)	9 (75%)	12	
	Total	17 (45%)	21 (55%)	38	0
Aphasia					
	Yes	10 (63%)	6 (37%)	16	
	No	6 (32%)	13 (68%)	19	
	Total	16 (46%)	19 (54%)	35	3
Mean tumor volume	In mm^3^	93,046.07	108,215.7		
Chemotherapy					
	Yes	11 (100%)	0 (0%)	11	
	No	5 (31%)	11 (69%)	16	
	Total	16 (59%)	11 (41%)	27	11
Radiotherapy					
	Yes	12 (100%)	0 (0%)	12	
	No	4 (27%)	11 (73%)	15	
	Total	16 (59%)	11 (41%)	27	11
Totals					27

## Data Availability

The authors carefully documented all methods, materials, and data that were used to conduct the research presented in this article, and they agree to share anonymized data upon request from any qualified investigator.
